# Drug Stability: ICH versus Accelerated Predictive Stability Studies

**DOI:** 10.3390/pharmaceutics14112324

**Published:** 2022-10-28

**Authors:** Olga González-González, Irving O. Ramirez, Bianca I. Ramirez, Peter O’Connell, Maria Paloma Ballesteros, Juan José Torrado, Dolores R. Serrano

**Affiliations:** 1 Departamento de Farmacia Galénica y Tecnología Alimentaria, Facultad de Farmacia, Universidad Complutense de Madrid, Plaza Ramón y Cajal, s/n, 28040 Madrid, Spain; 2 Instituto Universitario de Farmacia Industrial (IUFI), Facultad de Farmacia, Universidad Complutense de Madrid, Plaza Ramón y Cajal, s/n, 28040 Madrid, Spain

**Keywords:** stability, extemporaneous compounding, ASP

## Abstract

The International Council for Harmonisation of Technical Requirements for Pharmaceuticals for Human Use (ICH), along with the World Health Organization (WHO), has provided a set of guidelines (ICH Q1A-E, Q3A-B, Q5C, Q6A-B) intended to unify the standards for the European Union, Japan, and the United States to facilitate the mutual acceptance of stability data that are sufficient for registration by the regulatory authorities in these jurisdictions. Overall, ICH stability studies involve a drug substance tested under storage conditions and assess its thermal stability and sensitivity to moisture. The long-term testing should be performed over a minimum of 12 months at 25 °C ± 2 °C/60% *RH* ± 5% *RH* or at 30 °C ± 2 °C/65% *RH* ± 5% *RH*. The intermediate and accelerated testing should cover a minimum of 6 months at 30 °C ± 2 °C/65% *RH* ± 5% *RH* (which is not necessary if this condition was utilized as a long-term one) and 40 °C ± 2 °C/75% *RH* ± 5% *RH*, respectively. Hence, the ICH stability testing for industrially fabricated medicines is rigorous and tedious and involves a long period of time to obtain preclinical stability data. For this reason, Accelerated Predictive Stability (APS) studies, carried out over a 3–4-week period and combining extreme temperatures and *RH* conditions (40–90 °C)/10–90% *RH*, have emerged as novel approaches to predict the long-term stability of pharmaceutical products in a more efficient and less time-consuming manner. In this work, the conventional ICH stability studies versus the APS approach will be reviewed, highlighting the advantages and disadvantages of both strategies. Furthermore, a comparison of the stability requirements for the commercialization of industrially fabricated medicines versus extemporaneous compounding formulations will be discussed.

## 1. Introduction

Stability studies were designed for monitoring and evaluating the quality of Active Pharmaceutical Ingredients (API) and Finished Pharmaceutical Products (FPP) under the influence of different factors such as environmental conditions (temperature, moisture, light), API–excipients interactions, packaging materials, shelf life or container-closure systems during a certain period [1]. Even though regulatory requirements are also established to evaluate degradation products of the API and impurities, very little information is required regarding excipient stability in Annex 10 for stability testing of active pharmaceutical ingredients and finished pharmaceutical products [1], and this is a crucial point to take into consideration as it can also affect the quality of the formulation. Some excipients (e.g., glycerol) are prone to degradation during repeated use or improper storage, but impurities generated are not usually monitored but may affect drug stability [2]. The impact of water on the degradation of each type of solid dosage form differs completely. In some cases, the drug fully or partially dissolved, such as in solutions and suspensions. Drugs sensitive to hydrolysis are poor candidates for these types of liquid dosage forms. Regarding solid dosage forms, lyophilized products are more avid for water than tablets. The leftover water content in tablets after manufacturing is key, especially in moisture-sensitive drugs as it can accelerate drug degradation.

Stability is defined as the quality of being stable, and applying this wide concept into pharmaceutical formulations is considered as the absence of changes in characteristics and properties of the product at the time of its manufacture [3]. In general, five main facets determine stability: chemical, physical, microbiological, therapeutic, and toxicological. Drug substance attributes susceptible to change are the ones that should be evaluated; these conditions should be maintained throughout the shelf life of the product [3]. Depending on the dosage form, the instability can be manifested in diverse ways: drug precipitation, microbial contamination or chemical degradation for liquid dosage forms (solutions, elixirs), organoleptic changes in semisolid and solid dosage forms such as mottling and tackiness, as well as chemical issues such as hydrolysis and oxidation [3].

Chemical stability plays a major role in FPPs where the API is molecularly dispersed, such as in solutions, but also in semisolid and solid formulations. In high-water-content medicines, the evaluation of the microbiological stability is important as it can cause critical health care issues. Apart from potency loss and microbiological growth, degradants can appear over time and can lead to toxicity in patients, and hence, their quantification is key to determine the overall safety profile of the dosage form [4].

On the contrary, physical instability including appearance, palatability, uniformity, or dissolution mostly occurs in solid dosage forms as well as suspensions. The particle size of suspensions is a crucial factor to determine the biopharmaceutical performance of the formulation. Over time, the particle size growth can result in deflocculated suspensions; they are difficult to re-disperse, affecting the accurate dosing, but also, larger sizes can be detrimental to the oral drug bioavailability. Changes in physical stability are also fundamentally critical in amorphous solid dispersions. Over time, crystallization of the API can take place, and hence, this can cause a profound impact on the dissolution rate of the API and thus, its oral bioavailability [4].

Microbiological instability is a common factor of liquid dosage forms due to the high percentage of water of the preparation. To maintain the effectiveness, microbial growth is reduced by adding to the formulation of some antimicrobial excipients and using suitable storage conditions, as included in Table 1 [3].

## 2. Stability Studies

The stability paradigm was established by the publication of a set of stability guidelines (ICH Q1A-F, Q3A-B, Q5C, and Q6A-B) of APIs and FPPs in the early 2000s by the International Conference on Harmonization of Technical Requirements of Pharmaceuticals for Human Use (ICH) along with the World Health Organization (WHO). To unify the standards amongst the European Union, Japan, and the United States, and to define the core stability data storage required according to the climate zone (I–IV), a set of stability guidance was drafted and published to facilitate the mutual marketing authorization [6].

Following the requirements established by the ICH guidelines (40 °C ± 2 °C/75% *RH* ± 5% *RH* as general cases) for accelerated stability studies, it would take at least 6 months of experimental laboratory work to obtain preliminary stability data to include in a dossier that would be submitted to the competent regulatory medicine agencies. The development of novel tools to accelerate this process is crucial to ensure fast and correct decision-making by pharmaceutical companies during the preclinical phase development of new medicines. For example, different excipients can be selected during drug formulation development. Faster decisions to choose the right one should be made by attending to the in vivo drug performance but also drug stability. Accelerated Predictive Stability (APS) studies have been developed with this purpose and they can provide a data set in less than 1 month, hence saving time and reducing costs. The implementation of APS studies during the development of industrially fabricated medicines and extemporaneous compounding formulations will be discussed in this review.

Different storage requirements of temperature, humidity, and storing time are defined in the ICH guidelines for industrially fabricated medicines (APIs and FPPs) according to the type of study: long-term, intermediate and accelerated [1]. However, the stability of compounding preparations according to USP is not specifically described. It is based on the stability experience of compounds, limitation of the expiry date of each compounding monograph, and recommendations according to the dosage forms. The aim of the FDA is that pharmacists prepare extemporaneous compounded formulations when there is no suitable commercially available medicine in the market. Keeping a balance between suitability and stability is key when preparing extemporaneous formulations [7].

Stability studies are essential to ensure the quality, efficacy, and security of drug substances and products. These stability guidelines have the purpose to provide evidence of how the quality of the drugs is modified under different factors (light, humidity, and temperature) as well as the degradation processes that take place.

**Figure 1:**
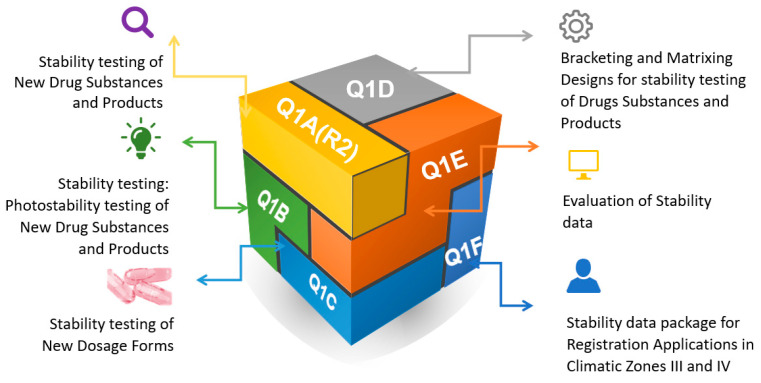
ICH codes and titles.

In the early 1990s, ICH guidelines compiled the steps to revise the stability data from industrially manufactured medicines. During the 2000s, The World Health Organization (WHO) made some modifications to these guidelines. Extreme climatic conditions for some countries were included, and also, defined guidelines for the global environment were added [8]. Information related to the stability data, the testing of new drug substances and products, the evaluation of the data, analysis of the impurities, and the storage conditions according to the climate zone were included and well defined in these guidelines (ICH Q1A-QF, 2003). The testing frequency, the storage conditions, the number of replicates, and the length of the studies should be defined in the dossier to cover the time of use, shipment, and storage (Figure 1) [9].

**Figure 2:**
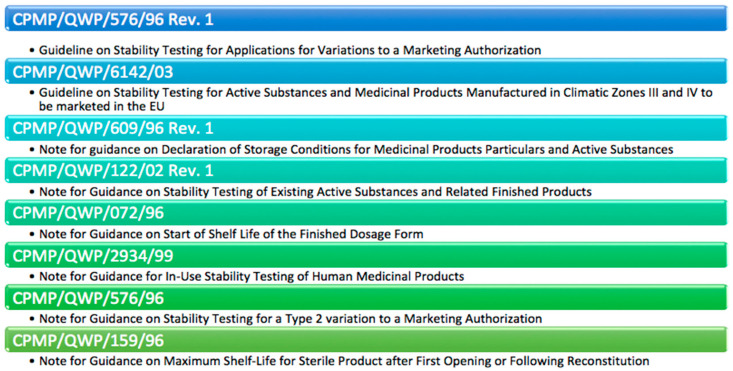
CPMP Guidelines. Modified from: [8].

Besides the ICH guidelines, the Committee for Proprietary Medicinal Products (CPMP) collects stability-testing rules (Figure 2). The European Agency for the Evaluation of Medicinal Products (EMEA) pursues the objective to support and hold pharmaceutical applications for the authorization of medicinal products in the European Union [10].

### Storage Conditions

For data submission to competent regulatory authorities, manufacturers should perform stability studies to ensure the storage, shipment, and conservation of FPPs during the whole product shelf life. Shelf life under different climate conditions should demonstrate thermal stability and sensitivity to moisture in the countries of destination. There are four climatic zones (I–IV) distinguished by their average temperature and humidity annual conditions. The climatic zones where the APIs or the FPPs are prescribed and sold determine the storage conditions that should be used [11]. ICH Q1A (R2) described the test conditions of stability studies agreed by Japan, the EU, and the United States for Climatic zones II and I. Stability information produced in any of these regions would be accepted by the rest and would be correctly labeled according to specific country requirements.

However, in 2003, ICH Q1F: Stability Data Package for Registration Applications in Climatic Zones III and IV was updated to include the countries not located in ICH Q1A (R2). ICH experts were forced to publish an explanatory note about the umbrella countries of the ICH and the agreed storage conditions to unify stability storage and study-length criteria and reduce the number of different storage conditions.

The consensual conditions were voted for by WHO through a survey amongst their member states to agree on the following conditions: 30 °C/65% *RH* as the long-term storage conditions for Climatic Zone III/IV countries [12,13].

**Figure 3:**
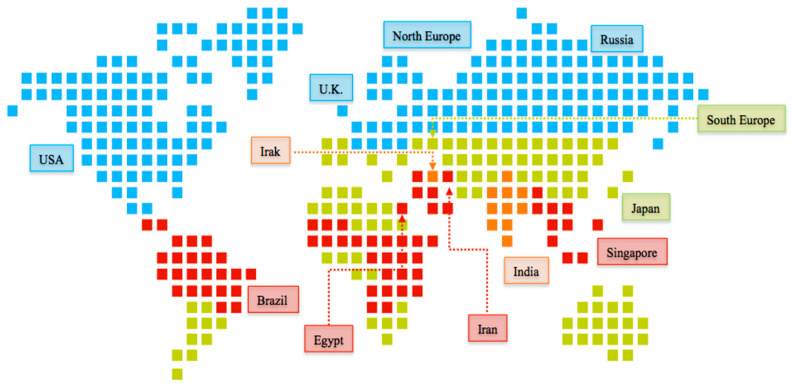
ICH climatic zones. Key: climate zone I (blue color), climate zone II (green color), climate zone III (orange color) and climate zone IVa and IVB (red color).

Nevertheless, some countries in Climatic Zone IV revised their stability guidelines and communicated the need to include a bigger margin of the percentage in *RH*, defining their long-term storage conditions as 30 °C/75% *RH*. Regulatory authorities have agreed to accept studies with more restricted humidity conditions [1,12] (Table 2, Figure 3).

The long-term (real-time) testing should include the duration of the submission of the primary batches and cover the retest period. During the period of registration, additional data from accelerated and intermediate studies can be used to evaluate the effect of excursions in extreme conditions outside the label storage recommendations (i.e., during shipment). Long-term storage should be predicted with the APS studies. If any excursions occurred during the testing in accelerated conditions, an explanation should be required to study the short-term excursions outside the label.

Another factor that should be controlled for its influence on the preparations is the container. The container closure system should be similar to the commercialized product and should include primary packaging components and secondary packaging components to protect the FPPs. Packaging materials are classified according to their permeability to moisture, light, and temperature [6,12] (Table 3).

## 3. APS Studies vs. ICH Studies

ICH guidelines have established the core of stability data required for selling APIs and FPPs and regulatory approval. Stability testing is based on several factors such as storage conditions, time of storing, and influence of environmental conditions or container closure systems. These storage conditions simulated in real-time the shipment, use of the product, and storage. According to the type of study, some steps and conditions are described for each type: long-term, intermediate, and accelerated studies (Table 3).

As a novel approach to predict the long-term stability of pharmaceutical products, Accelerated Predictive Stability studies (APSs) have emerged as a more efficient and less time-consuming approach. These studies are designed to increase the rate of pharmaceutical degradation and to measure the changes undergone by the products due to forced storage conditions.

**Figure 4:**
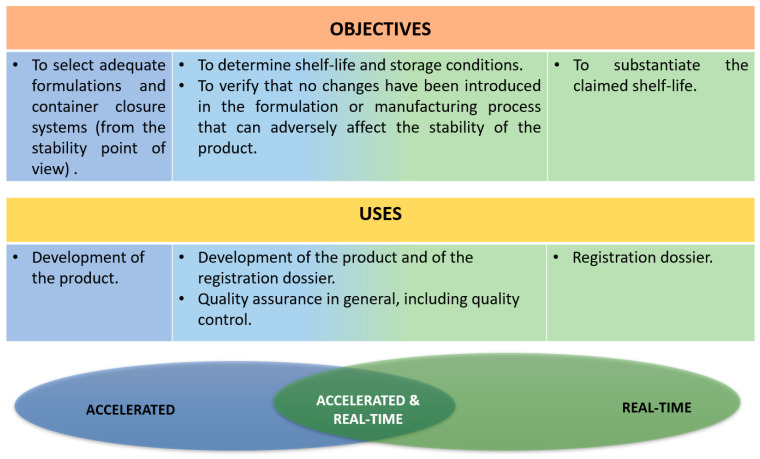
Objectives of Stability Testing. Adapted from: [8].

Depending on the type and purpose of stability studies, stability testing could be used for different applications: the development of the FPPs, the registration of the dossier, quality assurance, or quality control (Figure 4). These uses have the objective of selecting the most stable formulation and container closure, determining the shelf life and storage conditions, as well as verifying possible changes in the existing formulation [8].

## 4. Accelerated Predictive Stability (ASP) Studies

**Figure 5:**
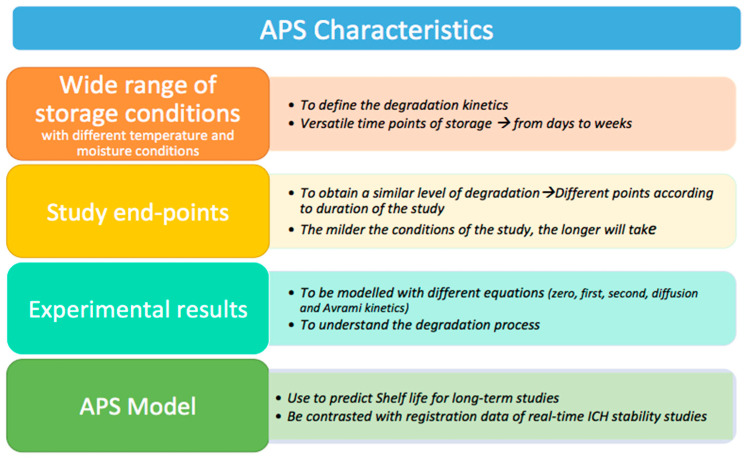
APS characteristics. Modified from: [16,17].

The accelerated stability assessment program (ASAP) was described to establish the stability modelling of pharmaceutical materials by Waterman and co-workers [15]. This program was based on a moisture-modified Arrhenius equation to provide a guiding protocol to follow when performing stability studies. Extreme temperatures and *RH* conditions (>40 °C) are combined during shorter periods (3–4 weeks) to understand which degradation kinetic governs the process and hence, to be able to extrapolate what happens under the conditions described in the ICH guidelines (Figure 5). These studies play an important role in understanding and assessing stability properties and advancing the pharmacokinetic behavior of drugs in the long term [16].

In contrast with ICH guidelines, APS focuses on reducing the time of testing (accelerated) and risk-analyses (predictive) and is defined by the following characteristics [16]:

- A wide range of storage conditions with different temperatures and moisture conditions are used to define the degradation kinetics. Time points of storage are versatile depending on the pharmaceutical stability, from days to weeks.
- Study endpoints are different according to the duration of the study to obtain a similar level of degradation. The milder the conditions of the study, the longer it will take.
- Experimental results are modelled using a range of different equations (e.g., zero, first, second, diffusion, and Avrami kinetics) to understand which kinetic model governs the degradation process.
- Shelf-life use is predicted for long-term studies using the APS model.
- APS models should be contrasted with registration data of real-time ICH stability studies.

This new tool, APS, can quickly and accurately predict the expiry date for APIs and FPPs, helping the decision-making process, which is especially critical in the pipeline when bringing new molecules and formulations to the market [15].

Different degradation kinetics are investigated during APS. These APS studies can predict excursions of the degradation and shelf life of long-term studies. The most influenced factors for the data analysis are internal factors such as product degradation and also external conditions (temperature, humidity, light, and oxygen concentration) [17].

### Application Areas of APSs

APS is considered a useful tool for defining and predicting the drug development life cycle, kinetic degradation, packaging selection, API retesting, and products of degradation, as well as understanding formulations [16,17]. APSs were developed to study a wide range of dosage forms (oral, topical, transdermal, or injectable forms) and APIs or container materials. They are also used to predict the degradation of products and to take advantage of suitable storage conditions for long-term studies. APSs follow Arrhenius kinetics to establish chemical and physical degradation of the active ingredients.

The application areas include clinical development (API selection, suitable packaging, storage condition specifications, shelf life, excipient election or equivalency of batch, supplier), applications during registration (supportive pharmaceutical development, QbD or package selection), and post-approval changes (assessment changes during evaluation about continent and container data).

## 5. Extemporaneous Compounding Formulations

The stability for compounding formulations is defined in the Pharmacopoeias and Nationals Formularies (NF). These documents compile the authorized monographs with the manufacturing conditions and storage specifications. Stability conditions for these formulations can usually differ from the industrially fabricated medicines.

Compounding guidelines are based on these reference documents and establish the stability of the preparation and the expiry date, according to the APIs, the container, and the law restrictions. Most of these formulations are manufactured in pharmacies or small laboratories without preserving excipients, following the health agencies’ recommendations and guidelines, and for this reason, the expiry date is more restrictive than industrially fabricated medicines.

To determine the exact expiry date of extemporaneous compounding formulations is a challenging task as it depends on the number of times that the container is opened during use, the dosage form, the hygiene of the patient, the storage, and handling conditions. Additionally, it is also important to consider the final characteristics of the formulation, especially how long the medication should be used for and also the presence of preservatives in the formulation, the material of the container employed, and the openness of the packaging.

As an example, we have taken into account the Spanish law, in which the validation period of extemporaneous compounding formulations is defined in monographs compiled in Nationals Formularies (RD 175/2001 of 23rd of February). Additionally, the expiry date documented in the literature is taken into account [18,19]. The recommended pharmaceutical validation period is mainly defined by the dosage form, the suitable storage temperature (room temperature: <25 °C), and the storage conditions recommended for dry and dark locations and hermetic containers [20].

**Figure 6:**
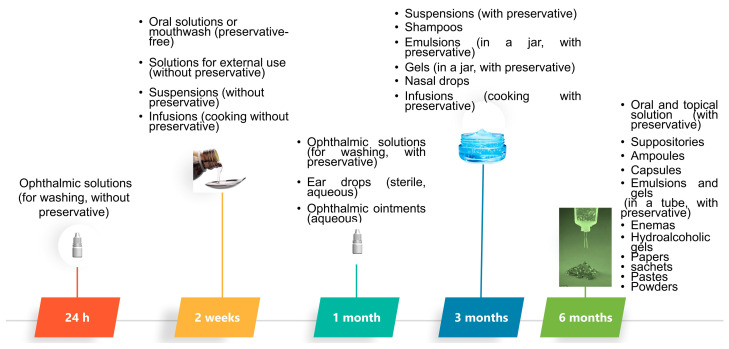
Recommended shelf life for extemporaneous compounding formulations. Modified from: [20].

In the case of aqueous solutions, the maximum time of shelf life is 6 months, for nose drops it is 3 months and for ophthalmic solutions it is 1 month, although it is important to remember that according to the Spanish law (RD 1718/2010 of 17th of December) about medical prescriptions and dispensing mediation, the expiry date should not exceed over 3 months (Figure 6) [21,22,23].

The USP and NF recommendations suggest a beyond-use time of 6 months (or no longer than 25% of the time remaining of the validation period) for non-aqueous liquids and solid formulations, 14 days for liquid preparations (stored between 2 and 8 °C) prepared in solid form and 30 days or no longer than the therapy duration for the rest of formulations. However, the beyond-use date could be extended with scientifically validated data [22].

Furthermore, certain compounding formulations can contain active ingredients with limited expiry dates, and for this reason, their period of validation is limited to 1 month. Some of these APIs require preservatives and antimicrobial excipients in their formulations, such as erythromycin, vitamin E, potassium permanganate, ascorbic acid, ketoconazole, and hydroxychloroquine.

## 6. Differences between APS and Forced Degradation Studies

Forced degradation studies are designed to identify most of the degradation products for an API, while APSs aim to predict the degradation kinetics and hence the shelf life of the product through the isoconversion time, which is defined as the time a certain degradation product, total degradants, or potency reach the specification limit [24]. The main objectives for forced degradation studies are [25]:

- To determine the structure of degradation products and their degradation reactions.
- To investigate the mechanism of the chemical degradation reactions, distinguishing between intrinsic degradation products from those related to the formulation.
- To reduce stability issues when formulating more stable preparations.
- To simulate products of degradation that can be formed under ICH conditions and should be quantified.

**Figure 7:**
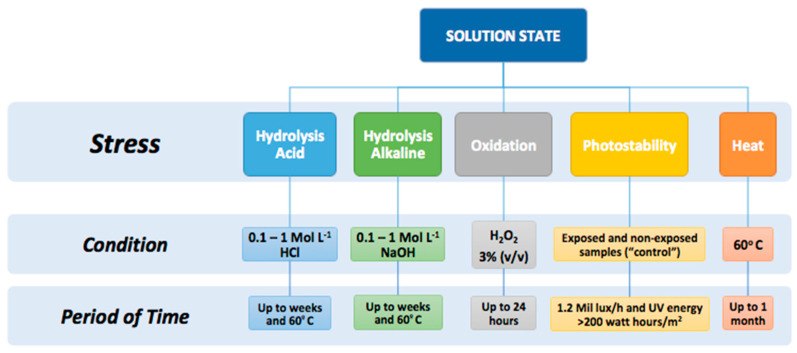
Forced degradation studies. Modified from: [24].

The existence of several types of degradation reactions promotes a huge variety of new structures (Figure 7). The most common degradation mechanism are oxidation, hydrolysis, thermal degradation, isomerization, and photolysis [4].

**Figure 8:**
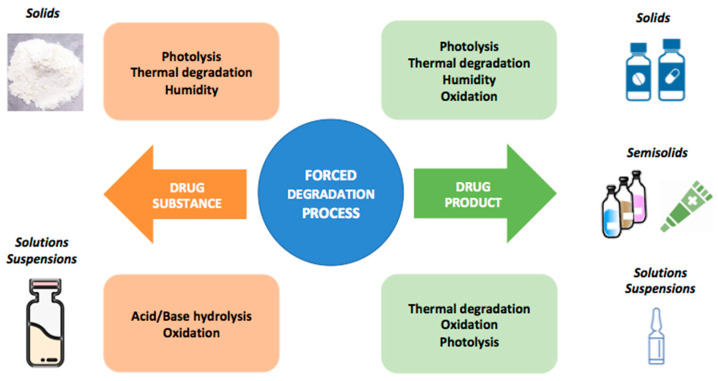
Used conditions for forced degradation studies. Modified from: [25,26,27].

There are certain functional groups located in the chemical structure of drugs that frequently suffer from hydrolysis (due to the presence of water molecules: H_3_O^+^, or OH^−^) (Figure 8). To prevent these reactions from occurring, the drug should be formulated at the highest stability pH by modifying the dielectric constant of the environment due to the addition of different cosolvents, by reducing the API solubility and decreasing its contact with water or by protecting the susceptible functional groups from the hydrolysis reactions [4].

Oxidation takes place when the pharmacological compound enters into contact with oxygen. Oxidation reactions tend to promote the formation of free radicals, which trigger chain reactions due to the electron transfer mechanism [4,25]. Photolysis is a kind of reaction experienced by certain drugs that are sensitive to sunlight, leading to the formation of degradation products. Some APIs can capture the UV light and become self-activated, leading to photolysis. Thermal degradation is performed in extreme heat conditions, not only under dry conditions but also with wet heat. The Arrhenius equation is the tool that allows us to study the impact of temperature on the activation energy (*Ea*) of the API [3,25].

## 7. Fundamentals behind an APS Study

APS aims to predict the stability of products in shorter periods using more extreme conditions. Temperatures commonly used range between 50 and 80 °C combined with different ranges of relative humidity (10–75%). In APSs, the most important factor is the isoconversion time, defined as the time to edge to failure, in other words, the time to reach a certain specification limit for potency or degradants. In traditional stability studies, degradation at fixed time points (i.e., 3, 6, 12, 18, 24 months) is determined and hence, there is a different level of degradation in each condition. However, in APS studies, the conversion to degradation products is kept constant to target the specification limit, while the time is varied at each condition [24].

Ideally, to estimate the isoconversion time with a small error, the degradation at a particular condition should be close to the specification limit. Hence, the different combinations of extreme relative humidity and temperatures should be carefully selected to avoid the need of a great extrapolation, which would results in lower precision. This implies prior knowledge of the API or formulation testing, which is not necessary for ICH studies [28,29].

Mathematical calculations in APSs are based on the use of the Arrhenius equation, which describes the relationship between the rate of degradation and temperature in a liquid state (Equation (1)) [29]:

(1) $$k=Ae^{-\frac{Ea}{RT}}$$

where *k* is the chemical reaction rate, *A* is the preexponential factor related to the probability of the molecules colliding with the correct orientation so that the collision gives rise to the product’s preexponential factor, *Ea* is the activation energy of the reaction, typically measured in kJ mol^−1^ or kcal mol^−1^, which describes the “temperature sensitivity” of the drug, *R* is the universal gas constant with values of 8.314 J K^−1^ mol^−1^ or 1.987 Cal K^−1^ mol^−1^ and *T* is the absolute temperature expressed in Kelvin degrees. According to the collision theory, when the temperature rises, molecules move faster and collide more vigorously, increasing the likelihood of bond cleavages and rearrangements. A high temperature and low activation energy favors larger degradation constants (k) and then speeds up the reaction. When temperature is raised, the number of molecules that possess an energy equal to or greater than the activation energy increases, which triggers the chemical reaction; in this case, it is drug degradation. From the exponential part of the Arrhenius equation (Equation (1)), the probability density that one model of a reactant has a kinetic energy above the activation energy can be known and hence, so can the likelihood for the reaction to occur.

Based on this premise, APSs performed at extreme temperatures will trigger the formation of degradation products faster. From a practical point of view, the linear form of this equation can be obtained by applying logarithms (Equation (2)) [30]:

(2) $$Lnk=LnA-\frac{Ea}{RT}$$

**Figure 9:**
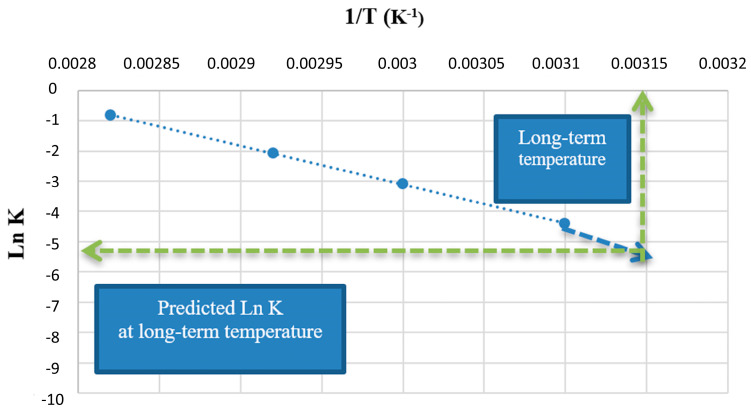
Drug shelf-life prediction derived from the extrapolation of degradation rates from the Arrhenius equation. Modified from [29].

The logarithmic form of the Arrhenius equation is used to easily represent the degradation rate in a straight line and to compare different degradation slopes. When representing *Lnk* in the ordinate axis and 1/*T* in the abscissa axis, experimental data follow a linear trend, which allows for extrapolating the *Lnk* that governs the degradation process at lower temperatures (Figure 9). Knowing the degradation rate at these temperatures (40 °C and below), a prediction of the shelf life at ICH conditions can be established.

However, pharmaceutical solid dosage forms are highly affected by moisture, and hence, the use of only the classical Arrhenius equation is not enough to make a good prediction of the shelf life. For this reason, another innovative concept behind APS is the use of the humidity-corrected Arrhenius equation, considering the effect of moisture (Equation (3)):

(3) $$Lnk=LnA-\frac{Ea}{RT}+B\left(RH\right)$$

where *B* is a coefficient that represents the “humidity sensitivity” of the drug and *RH* is the relative humidity expressed as a percentage (%). The humidity-corrected Arrhenius equation explains the influence of *RH* combined with temperature in drug degradation processes. Molecular mobility is also highly affected by relative humidity. Thus, the higher the values of *B*, the greater the impact of moisture on drug stability.

**Figure 10:**
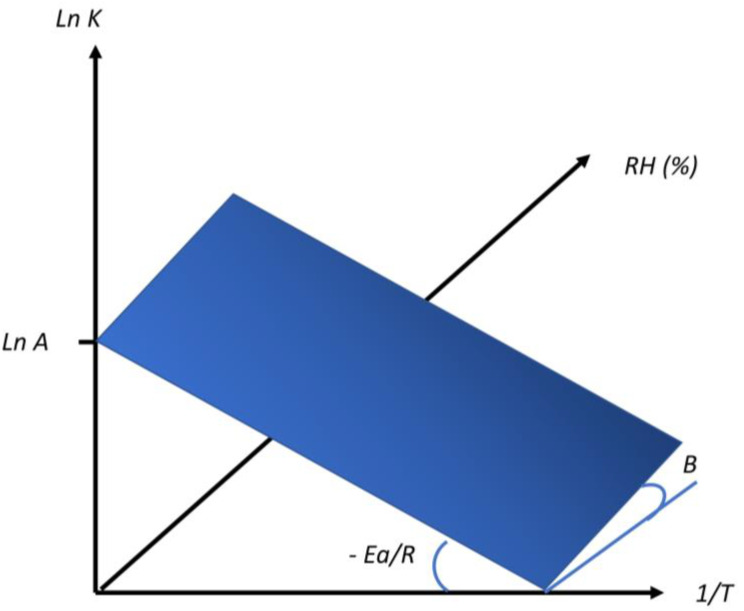
Three-dimensional representation of the degradation rate (*Lnk*), temperature (1/*T*) and relative humidity (%). Modified from [29].

The correlation amongst the rate of degradation (*Lnk*), relative humidity (%), and temperature (1/*T*) can be explained by a three-dimensional graph (Figure 10).

*Lnk* is represented in the abscissa axis, 1/*T* in the ordinate axis, and percentage of *RH* (% *RH*) in the z-axis, creating a plane in two dimensions. Lnk generates two slopes referring to the plane: the slope of Lnk against 1/*T* axis (represented in Figure 9 as: −*Ea*/R) and the slope of Lnk against % *RH* axis (represented in Figure 9 as coefficient B). Different software, such as ASAP Prime, can be used to extrapolate these values from the 3D graph [29]. The term B can be calculated using the following equation:

(4) $$B\left(RH\right)=Ln\frac{ln\;k1-k\;2}{RH1-RH2}$$

To enhance accuracy in the extrapolation of the term *B*, it is necessary to perform APS studies that include several conditions combining different *RH* values at the same temperature. In this case, the slope of *Lnk* against % *RH* can be calculated.

There is no mention in the ICH guidelines of the Arrhenius equation, while it exists in USP references [3,31,32] referring to the mean kinetic temperature (MKT). The MKT is the temperature calculated in a period as the sum of different degradations that happen at different temperatures. This temperature represents the storage effect and is calculated by the following equation:

(5) $$T_{k}=\frac{∆H/R}{-ln\left(\frac{e^{-\frac{∆H}{RT_{1}}}+e^{-\frac{∆H}{RT_{2}}}+\ldots +e^{-\frac{∆H}{RT_{n}}}}{n}\right)}$$

where the degradation rate (*k*) can be modified with temperature and humidity changes.

Determining the degradation rate (*k*) is very challenging and can raise multiple concerns. Different degradation models are expressed with a range of distinct profile graphics that can be linear or non-linear. According to the kinetics of the degradation process, several models of degradation have been defined (Table 4).

Linear degradation models are those employed to calculate the degradation rate (k) in which it is necessary to know the percentage of degradation and length of time-on-storage. Zero-order kinetics are those in which the degradation rate is unchanged as the amount of drug substance decreases [4,29]. However, it is relatively rare to find genuine zero-order reactions. Most zero-order reactions are actually first- or second-order reactions concerning the reactant, in which the reaction is stopped before the degradation rate starts to slow down due to consumption of the drug. Bearing in mind that specification limits for drug loss are commonly below 10%, greater percentages of degradation are not relevant, and thus we can assume that the drug substance degrades in an apparently linear fashion.

Non-linear degradation models are by far more common than linear ones. There are multiple reasons behind these profiles, such as (i) complex chemical degradation mechanisms in which many consecutive, competing, and reversible kinetics are involved, (ii) heterogeneous physical states in which each state may have different susceptibility to degradation, resulting in different fractions of reactive environments as time progresses, (iii) the limit of degradation, which is frequently below 100% and dictates the extent of the degradation process, e.g., when only a small fraction of the drug is in a reactive state, (iv) non-linear physical and solid-state processes such as nucleation, diffusion and contracting volume, and (v) non-fixed humidity environments, as the humidity inside the packaging can slowly increase due to moisture permeation through the packaging.

Based on the above circumstances, it is very challenging to determine accurately the value of “k” to model the effect of temperature and relative humidity on the drug substance stability. The degradation profile selected should represent what occurs at a lower extent of degradation, matching our specification limit. For this reason, the focus on the isoconversion time is key in the selection of the most suitable kinetic profile. The shape of the degradation curve does not matter, but that this shape remains consistent across all the different stability conditions tested does, and hence it can be determined how long our drug substance has to be exposed to a certain level of temperature and relative humidity for to reach the specification limit.

## 8. How to Implement an APS Study

Before running an APS protocol, careful considerations have to be taken into account to select suitable conditions of temperature and relative humidity. For example, the purpose of the study should be clear: is it to confirm long-term stability data or to verify what happens during an excursion at extreme conditions? Additionally, the specification limit for the API degradation and the ideal storage conditions for this product should be known (e.g., lyophilized products should not be exposed to extreme relative humidity conditions). Time points should be carefully chosen. Commonly, earlier time points are needed at greater temperature and relative humidity conditions. Finally, a suitable analytical test should be properly validated before starting the experiment to ensure high sensitivity and to obtain more accurate and reliable stability-prediction models.

After establishing the objective of an APS study, specific information about the API and the storage conditions should be compiled to elaborate on the protocol. Relevant physicochemical characteristics of the API should be studied, such as melting point, deliquescence, chances of crystallization if the drug is in the amorphous state, hydration and dehydration, and any other material attributes that may be relevant to set up the temperature and relative humidity conditions of the study.

Concerning the storage conditions, the temperature usually ranges from 50 to 90 °C; being the latter, the upper-temperature limit is commonly used to consider whether the vaporization of water molecules can take place at higher temperatures. The relative humidity is fixed in each stability chamber, ranging from 5 to 85%. Saturated salt solutions are used in APS studies to prepare a constant environment of *RH* in a closed container inside the chamber. The combination of temperature and relative humidity should be randomized to avoid alised conditions. A minimum of five conditions is recommended from a statistical point of view to maintain three degrees of freedom. Several time points, including repetitions, should be included in each condition to predict the degradation kinetics and establish a suitable relationship between time and degradation. Frequently, time points tend to be shorter (1–7 days) when using more extreme conditions (temperature above 70 °C and relative humidity above 50%) while longer time points (7–21 days) are selected at milder temperatures (below 70 °C) and relative humidity (<50%).

**Figure 11:**
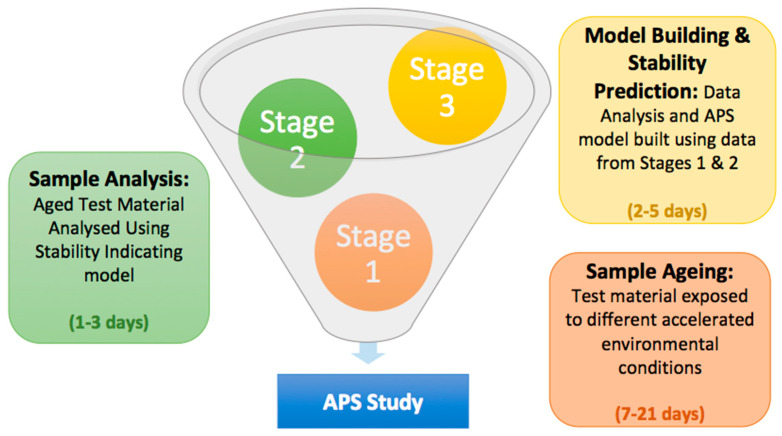
Schematic representation of the stages of an APS study.

In contrast with ICH guidelines, APS studies develop stability tests involving direct contact of the drug with temperature and relative humidity conditions inside stability chambers instead of performing the stability studies inside the packaging container. APS protocols commonly have three well-differentiated stages: sample ageing, sample analysis, and model building and stability prediction (Figure 11) [17].

Stage 1 (Sample Ageing): In this stage, the stability chambers are prepared based on the temperature and relative humidity conditions selected. Specific saturated salt solutions are introduced to each chamber to reach the desired relative humidity. Ideally, samples should not be introduced inside the chambers until the relative humidity and temperature have reached equilibrium, which should be confirmed with the corresponding sensor. Chambers with a small headspace tend to equilibrate faster than large containers. Once the stability chambers are ready, final dosage forms without packaging material are introduced in a glass vial within each chamber. Powder APIs can also be tested, for which it is recommended to weigh and introduce the same amount of material in each stability chamber. To minimize errors during the sample analysis at Stage 2, samples should be introduced sequentially in each chamber in such a way that at the end of the study all the samples are withdrawn and analyzed in a single batch.

Stage 2 (Sample Analysis): the aged test material will be analyzed using a stability-indicating HPLC method. Depending on the set APS protocol, the percentage of degradants or the drug loss at different time points and conditions will be quantified. It is key to ensure a high reproducibility in the method to reduce the overall standard error of the experiment.

Stage 3 (Model Building and Stability Prediction): the data obtained in stage 2 will be used to build the corresponding degradation kinetic models. The degradation rate will be determined based on the theoretical model that better fits the experimental data points in all the conditions tested. At this point, the isoconversion time should be considered and the model with the best fitting around the specification limit should be selected. Those conditions that led to a high percentage of degradation should be removed as they are not relevant and can alter the selection of the most suitable degradation profile. Once the degradation rates (“k”) are calculated, the activation energy and b value can be calculated from the modified Arrhenius equation. Based on this equation, the degradation rate at ICH conditions can be extrapolated, and hence, the long-term stability prediction can be obtained (Figure 11).

## 9. Stability Chambers

**Figure 12:**
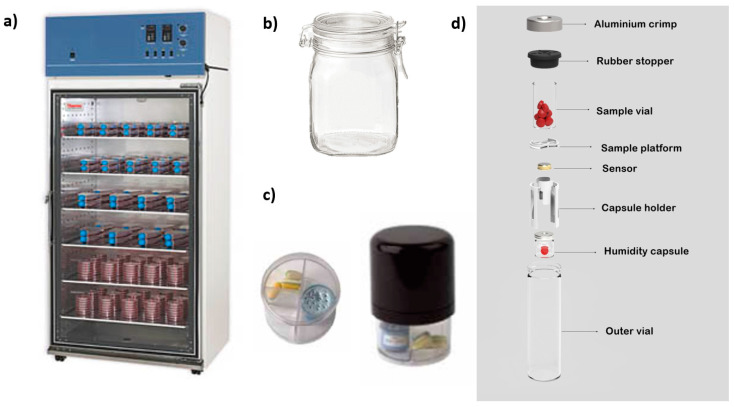
Types of stability chambers for ICH and APS studies. (**a**) Industrial stability chamber for ICH studies. (**b**) Simple hermetically closed jar for APSs, (**c**) Amebis Ageing System with WiFi controllers for APSs and (**d**) Cuspor Ageing System for APSs.

Storage conditions should be carefully monitored during the whole stability study in both ICH and APS studies. On certain occasions, it is difficult to reassure the maintenance of controlled temperature and humidity, especially when extreme conditions are used. There is a wide variety of stability chambers in the market. In ICH studies, large chambers with integrated temperature and moisture controllers are commonly used, with capacity for many samples (Figure 12a). On some occasions, large rooms are equipped with sensors to be used as stability chambers instead. However, the stability chambers for APSs are different, as a small number of samples and a greater number of conditions combining different relative humidity levels and temperatures are required. To meet this purpose, smaller containers are commonly utilized. These chambers can be just a hermetically closed glass container, such as basic jam jars (Figure 12b), or more advanced devices such as a Cuspor Ageing System (Figure 12c) or Amebis Stability Testing System (Figure 12d) [33,34], which include a sensor for both relative humidity and temperature and in which the empty headspace inside the chamber is limited to ensure that the relative humidity reaches a fast equilibrium after closure. To generate the relative humidity inside the smaller container, a saturated salt solution is used [35]. These systems have been successfully tested in a wide range of pharmaceutical stability studies [36,37,38,39].

## 10. Implementing APS Studies on Long-Term Dissolution Drug Performance

APSs have been successfully applied in the prediction of chemical stability; however, very little information can be found regarding their use in the prediction of physical changes such as color, hardness, disintegration, and dissolution [28]. Slowdown of dissolution performance on stability can potentially impact the drug bioavailability. The complexity is that the mechanisms of dissolution slowdown on stability are unclear, but as chemical degradation, temperature, relative humidity, and time are the key factors involved. The slowdown seems to occur by physical processes rather than chemical ones, due to a greater significance of the effect of relative humidity compared to temperature and the relative rapidity of the slowdown process.

Several authors have demonstrated the capability of APSs to predict not only the chemical stability but also the dissolution performance on stability [30,40]. When selecting the APS experimental conditions, the same principle as that for the chemical stability should be taken into consideration. For example, the conditions chosen should generate enough dissolution to slow down to reach the isoconversion (edge to failure) but not overly stress the product, as secondary mechanisms can occur. To build the models, the dissolution slows down and can be considered as potency loss over time. The data can still be fitted into the Arrhenius equation. The balanced residuals can be used as indicators of the accuracy of the prediction [41].

When predicting how the dissolution profile is affected during dissolution, a new parameter referred to as the acceleration factor can be used. This factor represents the degree by which the timescale of a dissolution profile needs to be scaled to overlay it onto the dissolution profile that was obtained initially (time 0). This factor follows an exponential decay curve under accelerated stability conditions. Although different dissolution rates are obtained at different conditions of temperature and relative humidity, the shape of the dissolution profile remains consistent in most cases, which allows extrapolation at ICH conditions [30].

## 11. Implementing APS on the Stability of Biological Products

According to ICH Q5C, the two key characteristics that should be included in the stability studies for biotechnological products are potency and purity; potency depends on the conjugation of active ingredients and purity is difficult to determine due to the effect of glycosylation or deamination. Other characteristics should also be monitored, such as closure system, additives, sterility, or visual appearance [42]. The testing frequency may differ from days to years to evaluate the real-time stability, and the long-term testing under extreme conditions is usually performed under different temperature conditions and protected from humidity.

The success of the APS methodology has promoted the desire to expand the use of these scientific concepts to other areas, such as biological products. However, additional considerations should be taken into account for these types of products, such as the physical conformation of the compound and its particle size, aggregation tendency, or denaturation. Conditions for APS studies should be carefully selected on a case-by-case basis [42,43,44].

Biological products are made up of active products with proteins and/or polypeptides in which the maintenance of the molecular conformation is quite characteristic and dependent on molecular forces. Complex analytical methods are needed to evaluate the stability of biological products (e.g., electrophoresis and size exclusion chromatography, besides those traditionally used such as HPLC, MS) as many external factors can influence them. It is important to consider that many of these products are thermodynamically unstable and the knowledge of the influence of temperature on their physical state is crucial to develop an APS study with a guarantee.

Despite the large number of investigations about the stability of small molecules (APIs and FPPs), the APS studies for peptides and biological products are very limited. Waterman and colleagues investigated the stability of bacitracin under APS studies using extreme temperatures from 50 to 80 °C and relative humidity up to 63% for 21 days [44]. The generated model’s predictions for the peptide were in agreement with long-term data at 30 °C/53% *RH* and 40 °C/75% *RH*, which validates this approach for accelerating the determination of the long-term stability of peptides [44]. Oliva and colleagues also developed an improved methodology to perform APS studies with peptide drugs. Combining the use of the reparametrized Arrhenius equation with the simultaneous treatment of all stability data using the bootstrap approach led to better long-term data prediction than the determination of individual rate constants [45]. However, more studies are still necessary to demonstrate the full potential and applicability of this approach in biological products.

## 12. Acceptability and Limitations of an APS Study

Accelerated stability studies have made great progress in predicting degradation models and reducing the costs of traditional stability studies according to ICH. However, when these two approaches are compared, the predictions do not always agree in terms of expected degradation [46].

These protocols are focused on the chemical degradation of both substances, APIs and FPPs, although neither physical aspects nor quality controls in solid products such as friability or hardness test of tablets are deeply examined. These limitations of APS are based on the incompatibility of physical changes and following the Arrhenius equation. Extreme temperatures should be used in APS studies; however, these temperatures should not exceed the melting point of the API or its deliquesce point. Similar considerations should be taken when testing amorphous solid dispersions, where selected temperatures below the glass transition of the amorphous system are recommended.

The acceptability of APS studies is based on their advantageous characteristics, as mentioned before. Their versatile time points of storage and points of analysis help us to study the pharmaceutical product during its whole life cycle. They offer suitable storage conditions, products of degradation, long-term shelf life, and an understanding of the degradation kinetics. All these reasons have converted APS into a key tool for stability studies, saving time and money and helping to evaluate the degradation of drugs in comparison to traditional ICH protocols, also providing the supportive data to predict traditional time-based long-term stability. In regulatory filings, even in some cases, they can be treated as the only data [47].

With all the progress made in this field, regulatory authorities such as FDA, Health Canada, and those from many European countries, are more frequently taking these APS protocols into consideration in the registration process of drugs, especially in the early phases of drug development (Phase I and Phase II) [48,49].

Some companies, such as Pfizer, have been utilizing risk tools as a design of experiment (DOE) based on the APS approach during the last decade. Robust protocol designs, storage conditions, the election of closure systems and the impact of changes in drug products settle the clinical and registration information to submit to authorities [49].

A new APS strategy is lean stability, a tool based on monitoring the relevant attributes and time points aimed at reducing the length of study and indeed determining shelf life. It includes technical adjustments to increase efficiency and assure the quality of the product, focusing on the individual product’s stability-related quality attributes. This alternate strategy allows companies to redirect their resources to these attributes and analyze historical data. APS studies use lean stability in unpacked drug product storage in different ranges of temperature and relative humidity to push the package material to its specification limits. Isoconversion allows us to extrapolate the shelf life to other conditions and is used with the moisture-modified Arrhenius equation for solid dosage forms [50,51].

## 13. Future Perspective and Conclusions

Pharmaceutical stability is an arduous concept to address considering its pleomorphic statuses, such as chemical, physical, and microbiological aspects. ICH guidelines dictate the standard conditions for performing long-term studies. Even though accelerated conditions are also proposed in the ICH guidelines, the time required to acquire all experimental data goes beyond at least 6 months, which is to the detriment of a fast decision-making and manufacturing process of industrially fabricated medicines. APS studies carried out over a 3–4-week period and combining extreme temperatures and *RH* conditions (>40 °C and up to 90% *RH*) have emerged as novel approaches to predicting the long-term stability of pharmaceutical products in a more efficient and less time-consuming manner.

The modification of the Arrhenius equation, including the moisture-sensitivity factor, has allowed us to take into account the impact of relative humidity on drug degradation, which is of special relevance when predicting the stability of solid dosage forms. However, the most important contribution of APS studies is the use of isoconversion time to predict stability, for which the conditions of relative humidity and temperature should be carefully selected to reach a certain specification limit for potency and degradants. The use of APS data is gaining importance as it can be used in the dossier submitted to regulatory authorities for drug approval. Apart from its usefulness in predicting chemical stability of small molecular weight drugs, other applications such as stability prediction of biological products and extrapolation of dissolution slowdown of solid dosage forms are under investigation, with promising results already having been obtained. In contrast, extemporaneous compounding formulations suffer from a very short shelf life, which is the consequence of the lack of scientific stability data to support longer storage periods. The application of a short APS protocol to extemporaneous compounding formulations could greatly benefit this type of medicine by extending their shelf life in a safer manner. This can be translated into a reduced waste of stable medicines and also a lower demand from hospital and community pharmacists to elaborate on this type of medicine.

## Figures and Tables

**Table 1:** Impact of drug instability in liquid formulations [4,5].

Type of Instability	Impact on Formulation Performance
Chemical	Loss of API content
	Appearance of drug degradation products or impurities
	Solvent evaporation resulting in an increase in API concentration
Physical	Lack of content uniformity
	Alteration of organoleptic properties leading to poor patient acceptability Crystallization of the API resulting in poor oral bioavailability
Biopharmaceutical	Alteration in drug conformation, bioavailability, and loss of activity
Microbiological	Contamination or increase in the number of microorganisms

**Table 2:** ICH Climatic zones and long-term stability conditions [6,9,14]. The minimum time duration for long-term studies is 12 months.

Climate Zone	Type of Climate	Countries	Long Term Testing Temperature (°C)	Long Term Testing Relative Humidity (*RH*)
I	Temperate	United Kingdom, Northern Europe, Russia, United States	21 °C	45%
II	Subtropical and Mediterranean	Japan, Southern Europe	25 °C	60%
III	Hot and dry	Iraq, India	30 °C	35%
IVa	Hot and humid	Iran, Egypt	30 °C	65%
IVb	Hot and very humid	Brazil, Singapore	30 °C	75%

**Table 3:** Types of stability studies for FPPs. Modified from: [6,12]. Key: RT, Room temperature.

Storage Condition	Container Type	Type of Study	ICH Storage Condition	Minimum Time Period Covered before Data at Submission
RT	General case	LONG-TERM	25 °C ± 2 °C/60% *RH* ± 5% *RH* or 30 °C ± 2 °C/65% *RH* ± 5% *RH* or 30 °C ± 2 °C/75% *RH* ± 5% *RH*	12 months or 6 months
		INTERMEDIATE	30 °C ± 2 °C/65% *RH* ± 5% *RH*	6 months
		ACCELE-RATED	40 °C ± 2 °C/75% *RH* ± 5% *RH*	6 months
	Impermeable containers	Packaging materials are classified depending on sealing, thickness, and permeability coefficients. In these packaging materials, the moisture sensitivity and risk of solvent loss is not critical. Stability studies can be performed under any controlled or ambient *RH* condition.		
	Semi-permeable containers	LONG-TERM	25 °C ± 2 °C/40% *RH* ± 5% *RH* or 30 °C ± 2 °C/35% *RH* ± 5% *RH*	12 months or 6 months
		INTERMEDIATE	30 °C ± 2 °C/35% *RH* ± 5% *RH*	6 months
		ACCELE-RATED	40 °C ± 2 °C/not more than (NMT) ± 25% *RH*	6 months
Refrigerated		LONG-TERM	5 °C ± 3 °C	12 months or 6 months
		ACCELE-RATED	25 °C ± 2 °C/60% *RH* ± 5% *RH* or 30 °C ± 2 °C/65% *RH* ± 5% *RH* or 30 °C ± 2 °C/75% *RH* ± 5% *RH*	6 months
Freezer		LONG-TERM	–20 °C ± 5 °C	12 months or 6 months

**Table 4:** Main kinetic models for degradation processes. Modified from: [17,29].

Name	Differential Form $\left(\frac{d\alpha }{dt}=\right)$
Zero-order	k
First-order	k(1−α)
Second-order	$k{\left(1-\alpha \right)}^{2}$
Power law (order “n”)	$k{\left(1-\alpha \right)}^{3}$
Nucleation (Avrami, order “n”)	$nk{\left(\alpha \right)}^{\left(n-1\right)/n}$
Contracting area (*n* = 2)/Contracting volume (*n* = 3)	$n\cdot k\left(1-\alpha \right){\left[-\ln\left(-\alpha \right)\right]}^{\left(n-1\right)/n}$
1-D diffusion	$n\cdot k{\left(1-\alpha \right)}^{\left(n-1\right)/n}$
Exponential	k·α

## Data Availability

No applicable.

## References

[1] WHO (2018). Annex 10. Stability Testing of Active Pharmaceutical Ingredients and Finished Pharmaceutical Products.

[2] Sun M.F., Liao J.N., Jing Z.Y., Gao H., Shen B.B., Xu Y.F., Fang W.J. (2022). Effects of polyol excipient stability during storage and use on the quality of biopharmaceutical formulations. J. Pharm. Anal..

[3] USP (2020). Stability Considerations in Dispensing Practice (1191). http://www.uspbpep.com/usp32/pub/data/v32270/usp32nf27s0_c1191.html#usp32nf27s0_c1191.

[4] Jato J.L.V. (2001). Estabilidad. Tecnologia Farmaceutica Volumen I: Aspectos Fundamentales de los Sistemas Farmacéuticos y Operaciones Básicas.

[5] Carstensen J.T. (2000). Drug Stability, Principles and Practices.

[6] ICH (2003). Q1A(R2): Stability testing of new drug substances and products. ICH Harmonised Tripartite Guideline Stability.

[7] Medisca Group (2017). Stability vs. suitability of compounded preparations: Customisation with appropriate compromise. Aust. J. Pharm..

[8] WHO (1996). Guidelines for Stability Testing of Pharmaceutical Products Containing Well Established Drug Substances in Conventional Dosage Forms.

[9] Singh S., Bakshi M. (2000). Guidance on Conduct of Stress Tests to Determine Inherent Stability of Drugs. Pharm. Technol. Asia.

[10] Bajaj S., Singla D., Sakhuja N. (2012). Stability Testing of Pharmaceutical Products. J. Appl. Pharm. Sci..

[11] Grimm W. (1986). Drugs Made in Germany.

[12] ICH (2003). Q1F: Stability Data Package for Registration Applications in Climatic Zones III and IV.

[13] ICH (1996). Q1C: Stability testing for new dosage forms. ICH Harmonised Tripartite Guideline Stability.

[14] Grimm W. (1998). Extension of the international conference on harmonization tripartite guideline for stability testing of new drug substances and products to countries of climatic zones III and IV. Drug Dev. Ind. Pharm..

[15] Waterman K.C., Carella A.J., Gumkowski M.J., Lukulay P., MacDonald B.C., Roy M.C., Shamblin S.L. (2007). Improved protocol and data analysis for accelerated shelf-life estimation of solid dosage forms. Pharm. Res..

[16] Qiu F. (2018). Accelerated Predictive Stability: An introduction. Accelerated Predictive Stability (APS) Fundamentals and Pharmaceutical Industry Practices.

[17] Qiu F. (2018). Practical considerations. Accelerated Predictive Stability (APS) Fundamentals and Pharmaceutical Industry Practices.

[18] SEFH: Sociedad Española de Farmacia Hospitalaria (2001). RD 175/2001, de 23 de Febrero, Sobre las Normas de Correcta Elaboración y Control de Calidad de Fórmulas Magistrales y Preparados Oficinales.

[19] Consejo General de Colegios Farmacéuticos (2017). Procedimiento de Formuación Magistral. Buenas Practicas en Farmacia Comunitaria.

[20] Colegio Oficial de Farmacéuticos de Granada, COFG (2009). Conservación de las fórmulas magistrales. Formulación Magistral: Normas de Calidad y Legislación.

[21] Glass B.D., Haywood A. (2006). Stability considerations in liquid dosage forms extemporaneously prepared from commercially available products. J. Pharm. Pharm. Sci..

[22] USP (2014). Pharmaceutical Compounding-Non Sterile Preparations (795). https://www.uspnf.com/notices/795-797-825-dependencies-20200424.

[23] SEFH: Sociedad Española de Farmacia Hospitalaria (2010). RD 1718/2010, de 17 de Diciembre, Sobre Receta Médica y Órdenes de Dispensación.

[24] Rack C. (2017). An Introduction to the Accelerated Stability Assesment Program (ASAP). Am. Pharm. Rev..

[25] Blessy M., Patel R.D., Prajapati P.N., Agrawal Y.K. (2014). Development of forced degradation and stability indicating studies of drugs—A review. J. Pharm. Anal..

[26] ICH (1996). Q1B: Stability testing: Photostability testing of new drug substances and products. ICH Harmonised Tripartite Guideline.

[27] Paola A., Tonhi E., Silv P., Shoyama Y. (2011). Stability Indicating Methods. Quality Control of Herbal Medicines and Related Areas.

[28] Qiu F., Wu Y., Hahn D., McMahon M., Orr R., Webb D., Hyzer C. (2017). Risk-Based Predictive Stability—An Industry Perspective. Pharm. Technol..

[29] Scrivens G., Clancy D., Gerst P. (2018). Theory and Fundamentals of Accelerated Predictive Stability (APS) studies. Accelerated Predictive Stability (APS) Fundamentals and Pharmaceutical Industry Practices.

[30] Scrivens G. (2019). Prediction of the Long-Term Dissolution Performance of an Immediate-Release Tablet Using Accelerated Stability Studies. J. Pharm. Sci..

[31] USP (2012). Pharmaceutical Stability (1050). Second Supplement to USP 35–NF 30.

[32] USP (2012). Good Storage and Distribution Practices (1079). Second Supplement to USP 35–NF 30 General Information.

[33] Cuspor Stability Experts Cuspor Ageing System. www.cuspor.com.

[34] Rotronic The Amebis Stability Testing System. https://www.instrumart.com/products/33267/rotronic-amebis-stability-testing-system.

[35] Young J.F. (1967). Humidity control in the laboratory using salt solutions—A review. J. Chem. Technol. Biotechnol..

[36] Rolon M., Hanna E., Vega C., Coronel C., Dea-Ayuela M.A., Serrano D.R., Lalatsa A. (2022). Solid Nanomedicines of Nifurtimox and Benznidazole for the Oral Treatment of Chagas Disease. Pharmaceutics.

[37] Cerda J.R., Arifi T., Ayyoubi S., Knief P., Ballesteros M.P., Keeble W., Barbu E., Healy A.M., Lalatsa A., Serrano D.R. (2020). Personalised 3D Printed Medicines: Optimising Material Properties for Successful Passive Diffusion Loading of Filaments for Fused Deposition Modelling of Solid Dosage Forms. Pharmaceutics.

[38] Serrano D.R., Fernandez-Garcia R., Mele M., Healy A.M., Lalatsa A. (2019). Designing Fast-Dissolving Orodispersible Films of Amphotericin B for Oropharyngeal Candidiasis. Pharmaceutics.

[39] Serrano D.R., Walsh D., O’Connell P., Mugheirbi N.A., Worku Z.A., Bolas-Fernandez F., Galiana C., Dea-Ayuela M.A., Healy A.M. (2018). Optimising the in vitro and in vivo performance of oral cocrystal formulations via spray coating. Eur. J. Pharm. Biopharm..

[40] Li H., Nadig D., Kuzmission A., Riley C.M. (2016). Prediction of the changes in drug dissolution from an immediate-release tablet containing two active pharmaceutical ingredients using an accelerated stability assessment program (ASAPprime®). AAPS Open.

[41] Qiu F., Scrivens G. (2018). ASAP Applications in clinical development: Prediction of Degradation and dissolution performance. Accelerated Predictive Stability (APS) Fundamentals and Pharmaceutical Industry Practices.

[42] ICH (2006). Q5C: Quality of Biotechnological Products: Stability Testing of Biotechnological/Biological Products.

[43] Frank F. (1994). Accelerated stability testing of bioproducts: Attractions and pitfalls. Trench Biotechnol..

[44] Waterman R., Lewis J., Waterman K. (2017). Accelerated Stability Modeling for Peptides: A Case Study with Bacitracin. AAPS PharmSciTech.

[45] Oliva A., Fariña J.B., Llabrés M. (2012). An improved methodology for data analysis in accelerated stability studies of peptide drugs: Practical considerations. Talanta.

[46] Williams H.E., Bright J., Roddy E., Poulton A., Cosgrove S.D., Turner F., Harrison P., Brookes A., MacDougall E., Abbott A. (2019). A comparison of drug substance predicted chemical stability with ICH compliant stability studies. Drug Dev. Ind. Pharm..

[47] Colgan S., Watson T., Whipple R., Nosal R., Beaman J. (2012). The application of science- and risk-based concepts to drug substance stability strategies. J. Pharm. Innov..

[48] Colgan S.T., Mazzeo T., Orr R. (2018). Regulatory expectations and industry practice on stability testing. Accelerated Predictive Stability (APS): Fundamentals and Pharmaceutical Industry Practices.

[49] RTimpano J., Freed A.L., Clement E., Masse K., Ryan K. (2018). Accelerated Predictive Stability (APS) Regulatory Strategies. Accelerated Predictive Stability (APS) Fundamentals and Pharmaceutical Industry Practices.

[50] Freed A.L., Colgan S.T., Kochling J.D., Alasandro M.S. (2017). AAPS Workshop: Accelerating pharmaceutical development through predictive stability approaches, April 4–5, 2016. AAPS Open.

[51] Colgan S., Hofer J., Timpano R., Vukovinsky K., Waterman K. (2015). Lean Stability. AAPS Mag..

